# Three *Yersinia enterocolitica* AmpD Homologs Participate in the Multi-Step Regulation of Chromosomal Cephalosporinase, AmpC

**DOI:** 10.3389/fmicb.2016.01282

**Published:** 2016-08-18

**Authors:** Chang Liu, Xin Wang, Yuhuang Chen, Huijing Hao, Xu Li, Junrong Liang, Ran Duan, Chuchu Li, Jing Zhang, Shihe Shao, Huaiqi Jing

**Affiliations:** ^1^Department of Pathogenic Biology, School of Medical Science, Jiangsu UniversityZhenjiang, China; ^2^National Institute for Communicable Disease Control and Prevention, Chinese Center for Disease Control and Prevention, State Key Laboratory of Infectious Disease Prevention and Control, Collaborative Innovation Center for Diagnosis and Treatment of Infectious DiseasesBeijing, China

**Keywords:** *Yersinia enterocolitica*, AmpC β-lactamase, AmpD, synergy effect, antimicrobial resistance

## Abstract

In many gram negative bacilli, AmpD plays a key role in both cell well-recycling pathway and β-lactamase regulation, inactivation of the *ampD* causes the accumulation of 1,6-anhydromuropeptides, and results in the *ampC* overproduction. In *Yersinia enterocolitica*, the regulation of *ampC* expression may also rely on the *ampR*-*ampC* system, the role of AmpD in this species is still unknown. In this study, three AmpD homologs (AmpD1, AmpD2, and AmpD3) have been identified in complete sequence of strain *Y. enterocolitica* subsp. *palearctica* 105.5R(r). To understand the role of three AmpD homologs, several mutant strains were constructed and analyzed where a rare *ampC* regulation mechanism was observed: low-effective *ampD2* and *ampD3* cooperate with the high-effective *ampD1* in the three levels regulation of *ampC* expression. *Enterobacteriaceae* was used to be supposed to regulate *ampC* expression by two steps, three steps regulation was only observed in *Pseudomonas aeruginosa*. In this study, we first reported that *Enterobacteriaceae Y. enterocolitica* can also possess a three steps stepwise regulation mechanism, regulating the *ampC* expression precisely.

## Introduction

*Yersinia enterocolitica* is a human enteric pathogen with worldwide distribution. It is a highly heterogeneous species with six biovars (1A, 1B, 2, 3, 4, and 5) that has more than fifty serotypes with different geographical distribution, ecological niches, and pathogenic properties (Wang et al., 2009; Liang et al., 2012). Most *Y. enterocolitica* strains produce two kinds of chromosomal β-lactamases, BlaA (a non-inducible broad-spectrum carbenicillinase) and BlaB (an AmpC-type inducible group I class C cephalosporinase; Cornelis and Abraham, 1975). A recent study shows in 1B/O:8 strain 8081, the expression of BlaA is constitutive whereas the AmpC (BlaB) was inducible (Bent and Young, 2010). Meanwhile, the two β-lactamases are not expressed simultaneously in all strains because strains with different biotypes tend to display specific resistance phenotypes (Stock et al., 2000). The mechanism of variable expression and activities of these two β-lactamases is unknown (De La Prieta et al., 1995, 2006; Stock et al., 1999; Pham et al., 2000; Sharma et al., 2006).

Chromosomal cephalosporinase AmpC is ubiquitous in Gram-negative organisms (Jacoby, 2009). Most strains express the AmpC β-lactamase constitutively at a low basal level and expression is high in induced conditions. The regulation of *ampC* expression is controlled by several regulatory genes, e.g., *ampR, ampG*, and *ampD*, which belong to the *ampR-ampC* system as part of the cell-wall recycling pathway (Park and Uehara, 2008). Bacterial peptidoglycan is remodeled in a dynamic balance between synthesis and degradation. During growth, the peptidoglycan in the periplasm is hydrolyzed by murein hydrolases to generate peptidoglycan (PG) degradation products (GlcNAc-1,6-anhydro-MurNAc; Johnson et al., 2013). The products are transported to the cytosol via an inner membrane permease, AmpG (Cheng and Park, 2002; Park and Uehara, 2008; Johnson et al., 2013), playing roles for cell wall precursors (Park and Uehara, 2008) and signaling molecules for the induction of β-lactamase (Jacobs et al., 1994; Hanson and Sanders, 1999). AmpD is a cytoplasmic N-acetyl-anhydromuramyl-L-alanine amidase, which has been well-studied in *Enterocobacter cloacae, Citrobacter freundii*, and *Pseudomonas aeruginosa*. GlcNAc-1,6-anhydromuropeptide and 1,6-anhydromuropeptide are hydrolyzed by AmpD to generate 1,6-anhydromuramic acid and peptide where the peptide is reused by enzymes in the cell wall recycling pathway to generate UDP-MurNAc-pentapeptide that binds to the transcriptional regulator AmpR allosterically to repress *ampC* expression (Lindberg et al., 1987; Holtje et al., 1994; Jacobs et al., 1997).

Inactivation of AmpD is the major cause of constitutive hyper-production of AmpC giving high β-lactam resistant phenotypes (Peter et al., 1988; Jacobs et al., 1995). In a recent study, Juan et al. demonstrated a stepwise upregulation mechanism with three AmpD homologs termed AmpD, AmpDh2, and AmpDh3; a highly sophisticated regulation mechanism for *ampC* expression in *P. aeruginosa* PAO1, the first characterized example in a multiple-step sequential regulation of β-lactamase expression in Gram-negative bacteria (Juan et al., 2006). Further, after the discovery of AmpD-like lipoprotein, AmiD, in *Escherichia coli* (Kerff et al., 2010), *Enterobacteriaceae* were thought to have *ampD* homologs (Juan et al., 2006; Yang et al., 2009), regulating *ampC* expression in two steps, however, there is no structural data are available. *Y. enterocolitica* was supposed to regulate *ampC* expression by *ampC-ampR* system because the presents of *ampC-ampR* region in chromosome (Seoane et al., 1992), but the feature of *ampC* regulation in *Y. enterocolitica* is still a mistiness. In the present study, we found *Y. enterocolitica* strain subsp. *palearctica* 105.5R(r) had three putative *ampD* homologs. This suggested *Y. enterocolitica* possessed a complex regulation mechanism for β-lactamase expression.

## Materials and methods

### Bacterial strains and growth conditions

The bacterial strains used in this study were listed in Table 1. The wild-type *Y. enterocolitica* strain subsp. *palearctica* 105.5R(r) (bioserotype 3/O:9) chromosome was completely sequenced as described in our previous work (Wang et al., 2011). Strains were routinely grown in Luria Bertani (LB) broth or on LB plates at 28°C (*Y. enterocolitica*) and 37°C (*E. coli*). When appropriate, antibiotics were added to the media as required to a final concentration of 34 μg/ml for chloramphenicol (Cm) and 5 μg/ml for tetracycline (Tc). *Yersinia* selective supplement was added to the solid LB medium as suggested by the manufacturer (Oxoid, UK).

**Table 1:** **Strains and plasmids used in this study**.

**Strains or plasmid**	**Genotype or relevant characteristics**	**Source or References**
***Yersinia enterocolitica***		
105.5R(r)	Wild type; completely sequenced	Wang et al., 2011
YEΔD1	105.5R(r) Δ*ampD1*	This work
YEΔD2	105.5R(r) Δ*ampD2*	This work
YEΔD3	105.5R(r) Δ*ampD3*	This work
YEΔD1D2	105.5R(r) Δ*ampD1* Δ*ampD2*	This work
YEΔD1D3	105.5R(r) Δ*ampD1* Δ*ampD3*	This work
YEΔD2D3	105.5R(r) Δ*ampD2* Δ*ampD3*	This work
YEΔD1D2D3	105.5R(r) Δ*ampD1* Δ*ampD2* Δ*ampD3*	This work
105.5R(r)-*ampC*Lux	105.5R(r) containing plasmid pBBR-*ampC*-Lux	This work
YEΔD1-*ampC*Lux	YEΔD1 containing plasmid pBBR-*ampC*-Lux	This work
YEΔD2-*ampC*Lux	YEΔD2 containing plasmid pBBR-*ampC*-Lux	This work
YEΔD3-*ampC*Lux	YEΔD3 containing plasmid pBBR-*ampC*-Lux	This work
YEΔD1D2-*ampC*Lux	YEΔD1D2 containing plasmid pBBR-*ampC*-Lux	This work
YEΔD1D3-*ampC*Lux	YEΔD1D3 containing plasmid pBBR-*ampC*-Lux	This work
YEΔD2D3-*ampC*Lux	YEΔD2D3 containing plasmid pBBR-*ampC*-Lux	This work
YEΔD1D2D3-*ampC*Lux	YEΔD1D2D3 containing plasmid pBBR-*ampC*-Lux	This work
***E. coli***		
DH5α	F-*endA1 hsdR17* (rk−, mk+) *supE44 thi-1 λ-recA1 gyrA96 relA1 deoR* Δ*(lacZYA-argF)*-U169 * 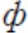 80dlac*ZΔM15	Invitrogen
S17 λpir	λ-pir R6K(*thi thr leu ton lacY supE recA*::RP4-2Tc::Mu)	Simon et al., 1983
**PLASMIDS**		
pDS132	CmR; Conditionally replicating vector; R6K origin, *mobRK4* transfer origin, sucrose-inducible *sacB*	Philippe et al., 2004
pDSD1	CmR; pDS132 containing 5′ and 3′ flanking sequence of amp*D1*	This work
pDSD2	CmR; pDS132 containing 5′ and 3′ flanking sequence of amp*D2*	This work
pDSD3	CmR; pDS132 containing 5′ and 3′ flanking sequence of amp*D3*	This work
pBBRLux	CmR; Luminescence without promoter (or contains a promoterless *luxCDABE* reporter)	Zhou et al., 2013
pLUX*ampC*	CmR; pBBRlux containing 250 bp 5′ flanking sequence of *ampC*	This work
pSRKTcD1	TcR; pSRKTc containing 105.5R(r) *ampD1* gene	This work
pSRKTcD2	TcR; pSRKTc containing 105.5R(r) *ampD2* gene	This work
pSRKTcD3	TcR; pSRKTc containing 105.5R(r) *ampD3* gene	This work

#### Construction of *ampD1, ampD2*, and *ampD3* mutant strains

After a homology search, we identified three potential *Y. enterocolitica ampD* homologs termed *ampD1, ampD2*, and *ampD3*. To further study them, three *ampD* single (YEΔD1, YEΔD2, YEΔD3), three double (YEΔD1D2, YEΔD1D3, YEΔD2D3), and one triple (YEΔD1D2D3) mutants were constructed using the following steps. Briefly, to three single *ampD* mutant strains, two PCR amplicons (Table 2) upstream and downstream of *ampD1, ampD2*, and *ampD3* were cloned into the suicide plasmid, pDS132 (Philippe et al., 2004), using restriction with *SphI* and *SacI* with the in-fusion cloning technique to obtain plasmids pDSD1, pDSD2, pDSD3. Recombinant plasmids were then transformed into *E. coli* DH5α and transformed into the helper strain, S17 λpir (Simon et al., 1983); and selected on 34 μg/ml chloramphenicol LB agar plates. Using conjugation, recombinant plasmids were introduced into *Y. enterocolitica* 105.5R(r); and transconjugants were selected using LB plates with 34 μg/ml chloramphenicol in Yersinia selective agar medium (Oxoid) and then incubated on LB plates without antibiotic overnight. For mutant selection, bacterial cultures were transferred onto LB plates containing 10% sucrose without NaCl. The mutants were confirmed using antibiotic resistance, PCR amplification, and sequencing. The double mutants and triple mutant strains were then constructed from the single mutants sequentially using the same procedures.

**Table 2:** **Primers used in this work**.

**Primer**	**Sequence (5′–3′)**	**PCR product size (bp)**	**Use**
pΔ*ampD1*U-F	GAGGTACCGCATGGCCTGTTTCAGCATAGTTGC	952	*ampD1* inactivation
pΔ*ampD1*U-R	ACAAAGTGACAAACTATACGTTACCTAAGCCCCCTAACCT		
pΔ*ampD1*D-F	AGGTTAGGGGGCTTAGGTAACGTATAGTTTGTCACTTTGT	945	
pΔ*ampD1*D-R	GAATTCCCGGGAGGCACCATAAATAGTCAGTAA		
pΔ*ampD2*U-F	GAGGTACCGCATGTACAAGCATTGGGTGAAGAA	975	*ampD2* inactivation
pΔ*ampD2*U-R	TTAAATAACTTTTACCGCGCAAGCACAGTTATAGTGAACC		
pΔ*ampD2*D-F	GGTTCACTATAACTGTGCTTGCGCGGTAAAAGTTATTTAA	992	
pΔ*ampD2*D-R	GAATTCCCGGGAGGTAACTGACCTGACCGTTCC		
pΔ*ampD3*U-F	GAGGTACCGCATGTTTATCGACACTCACAACTA	954	*ampD3* inactivation
pΔ*ampD3*U-R	TGGCGGCGCTGTATCTAGTCTAATGTTATTTATTGAGGAT		
pΔ*ampD3*D-F	ATCCTCAATAAATAACATTAGACTAGATACAGCGCCGCCA	945	
pΔ*ampD3*D-R	GAATTCCCGGGAGGTATCAGCCAATCACCAATG		
pP*ampC*-F	CGAGCTCGCGTATCCGCGATAC	384	*ampC* promoter activity
pP*ampC*-R	CGGATCCTAGTAAATCTTCCAT		
pC*ampD1*-F	CCATATGATGCAGTTAGAAAATAACTG	576	*ampD1* complementation
pC*ampD1*-R	CGAGCTCTTACGATGGTAAAGATGACT		
pC*ampD2*-F	CCATATGATGAGGAAGTTATTAAGCAC	852	*ampD2* complementation
pC*ampD2*-R	CGAGCTCTCAAGGATGAAGGGGACGAT		
pC*ampD3*-F	CCATATGATGTATATGATTGACTATAA	777	*ampD3* complementation
pC*ampD3*-R	CGAGCTCCTAGTTCTGTGCCGGAAAAT		

*Restriction enzyme recognition sites are underlined*.

### Antibiotic susceptibility testing

The minimum inhibitory concentrations (MICs) of 15 antibiotics were determined in triplicate using the standard 2-fold serial broth microdilution method according to the guidelines of the Clinical Laboratory Standards Institute (CLSI, 2015) for ampicillin, ticarcillin, cefazolin, piperacillin, ceftazidime, cefriaxone, cefepime, piperacillin/tazobactam, ampicillin/sulbactam, cefoxitin, cefotetan, aztreonam, imipenem, meropenem, and ciprofloxacin. *Escherichia coli* ATCC 25922 was used as a control strain in each assay.

### Measurement the expression of *blaA* and *ampC* using *luxCDABE* reporter system

The expression levels of *blaA* and *ampC* were determined using the *luxCDABE* reporter system. Primers pP*ampC*-F, pP*ampC*-R were used to amplify the *ampC* promoter region fragment, respectively; and was cloned into the plasmid pBBRLux (Hammer and Bassler, 2007) which carried promoter-less *luxCDABE* genes to generate the fusion plasmid pLUX*ampC*. The resulting plasmid was then introduced into *E. coli* S17 λpir and finally transferred into the wild-type strain 105.5R(r) and the seven derivate *ampD* mutants with or without cefoxitin. The inducibility of BlaA and AmpC β-lactamase was confirmed. Overnight cultures were diluted 1:100 into 15 ml LB broth and incubated with shaking at 28°C until mid-log phase was attained, then the cultures were incubated with the presence of 1/4- to 10-fold of MIC-values of imipenem and cefoxitin for an additional 1 h before harvesting. Luminescence was measured using an Infinite M200 Pro spectrophotometer and calculated with relative light units. Mean luminescence/OD600-values were obtained in three independent experiments (Zhou et al., 2013).

### Quantification of β-lactamase activity

β-Lactamase specific activity was determined spectrophotometrically in crude sonic extracts from strain 105.5R(r) and the seven above-described *ampD* mutants. To determine the β-lactamase specific activity after induction, the cultures were incubated in the presence of 40 μg/ml cefoxitin for 1 h before harvesting. The samples were centrifuged for 5 min at 3000 × g and the pellets were washed once in 5 ml 0.01 M phosphate buffered saline, pH 7.0 and re-suspended in 1 ml of the same buffer. The suspensions were sonicated on ice for 3 min, and then centrifuged at 10,000 × g for 30 min at 4°C and the supernatant was retained. After determining the total protein using Bio-Rad protein assay reagents, 10 min reactions were allowed before nitrocefin hydrolysis was measured. The specific activity (U/mg) was calculated as nanomoles of nitrocefin hydrolyzed per minute per milligram of protein, using an extinction coefficient (Δε) of 20,500 M^−1^ cm^−1^ for nitrocefin at 486 nm, as suggested by the manufacturer (Oxoid, UK; Kong et al., 2005). The mean β-lactamase activity obtained in three independent experiments was analyzed.

### Complementation assay

In the complementation assay, the ORFs of *ampD1, ampD2*, and *ampD3* were amplified and cloned into the broad-host-range expression vector, pSRKTc, to generate pSRKTcD1, pSRKTcD2, and pSRKTcD3, respectively. The three recombinant plasmids and plasmid pSRKTc (as a control) were transferred into the completely de-repressed strain YEΔD1D2D3. Transformants were selected on 5 μg/ml tetracycline Yersinia selective plates. Finally, β-lactamase activity was determined to evaluate the function of each AmpD homolog.

## Results

### *Yersinia enterocolitica* subsp. *palearctica* 105.5R(r) has three AmpD homologs

**Figure 1:**
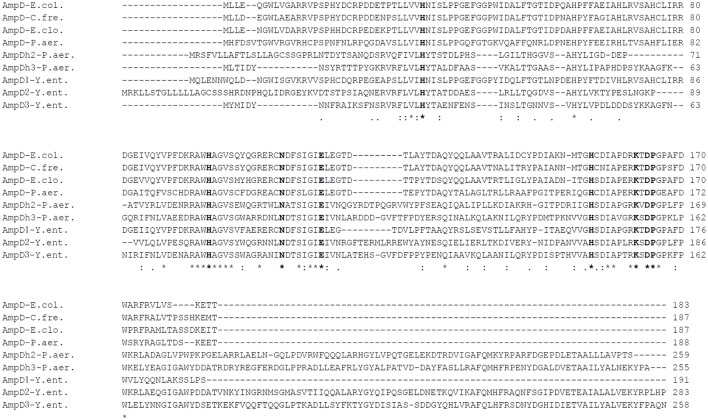
**Amino acid sequence alignment of AmpD homologs of different Gram-negative bacteria using ClustalW**. AmpD-E.col (*E. coli* K-12 accession no. AAC73221), AmpD-C.fre (*C. freundii* OS60 accession no. Z14002), AmpD-E.clo (*E. cloacae* accession no. CAA78391), AmpD-P.aer (*P. aeruginosa* PAO1 accession no. NP_253211), AmpDh2-P.aer (*P. aeruginosa* PAO1 accession no. NP_254172), AmpDh3-P.aer (*P. aeruginosa* PAO1 accession no. NP_249498), AmpD1-Y.ent (*Y. enterocolitica* accession no. WP_005156822), AmpD2-Y.ent (*Y. enterocolitica* 105.5R accession no. WP_005164953) and AmpD3-Y.ent (*Y. enterocolitica* 105.5R accession no. WP_013649890) are shown. Asterisk, colons, and periods represent identical, conserved, and semi-conserved residues, respectively. The conserved and essential amino acids for AmpD activity are indicated in bold.

**Figure 2:**
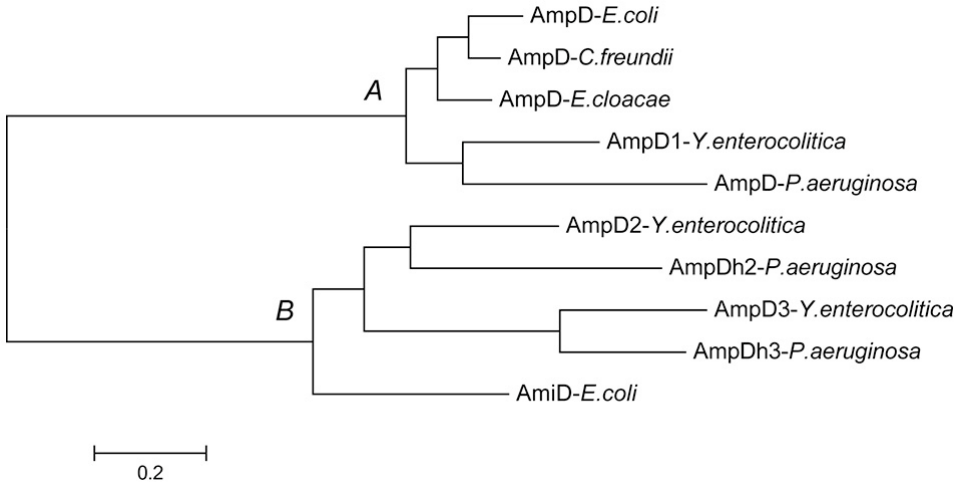
**Amino acid sequence phylogenetic analysis of AmpD homologs from different Gram-negative bacteria**. *Y. enterocolitica* AmpD1 belong to the branch A with the other “traditional” AmpD, *Y. enterocolitica* AmpD2 and AmpD3 belong to the second branch with *P. aeruginosa* AmpDh2 and AmpDh3 discovered recently.

As shown in Figure 1, the predicted amino acid sequences of AmpD were screened using ClustalW software multiple sequence alignment of AmpD1 (accession no. WP_005156822), *Y. enteoroclitica* AmpD2 (accession no. WP_005164953), AmpD3 (accession no. WP_013649890), and other closely related bacterial strains e.g., the AmpD from the *E. coli* K-12 (accession no. AAC73221), AmpD of *C. freundii* OS60 (accession no. Z14002), AmpD of *E. cloacae* ATCC13047 (accession no. CAA78391) and the AmpD (accession no. NP_253211), AmpDh2 (accession no. NP_254172), and AmpDh3 (accession no. NP_249498) of *P. aeruginosa* PAO1. The predicted amino acid sequence for the AmpD1 protein exhibited 76.4, 76.1, 76.7, and 70% identity to the AmpD of *E. coli* K-12, *C. freundii* OS60, *E. cloacae* ATCC13047, and *P. aeruginosa*, respectively. Further, AmpD2 (AmpD3) exhibited 44.9% (38%) and 44.8% (65.2%) identity to *P. aeruginosa* AmpDh2 and AmpDh3, respectively. All three AmpD homologs in *Y. enterocolitica* have four common residues at positions 34(H), 69(H), 154(H), and 165(P) shown by Jacobs et al. (1995); and six essential residues 34(H), 116(E), and 154(H), 162(K), 164(D) for *C. freundii* AmpD activity reported by Genereux et al. were also found (Genereux et al., 2004). The Amino acid phylogenetic trees of AmpD formed two branches: branch A contained *Y. enterocolitica* AmpD1 and all other “traditional” AmpD from different strains; branch B contained AmpD2, AmpD3 from *Y. enterocolitica* and AmpDh2 and AmpDh3 from *P. aeruginosa* PAO1 (Figure 2). Distribution of the three AmpD homologs in *Y. enterocolitica* was different. Using the DNA BLAST program provided by NCBI, we found the *ampD1* and *ampD2* were widely distributed in the *Y. enterocolitica* strains including 8081 (O:8), WA (O:8), Y11 (O:9), W22703 (O:9), Y53/03(O:5), and even in the atypical strain LC20. However, only three *Y. enterocolitica* strains (2015-87, W22703, Y11) and 105.5R(r) have *ampD3* genes among the available uploaded DNA sequences. According to the SignalP4.1 Server prediction (Petersen et al., 2011), the AmpD1 and AmpD3 protein is likely a cytoplasmic protein, AmpD2 is likely a secretory protein with signal peptide sequence.

### AmpD1, AmpD2, and AmpD3 are negative regulators of *ampC* expression at different levels

**Figure 3:**
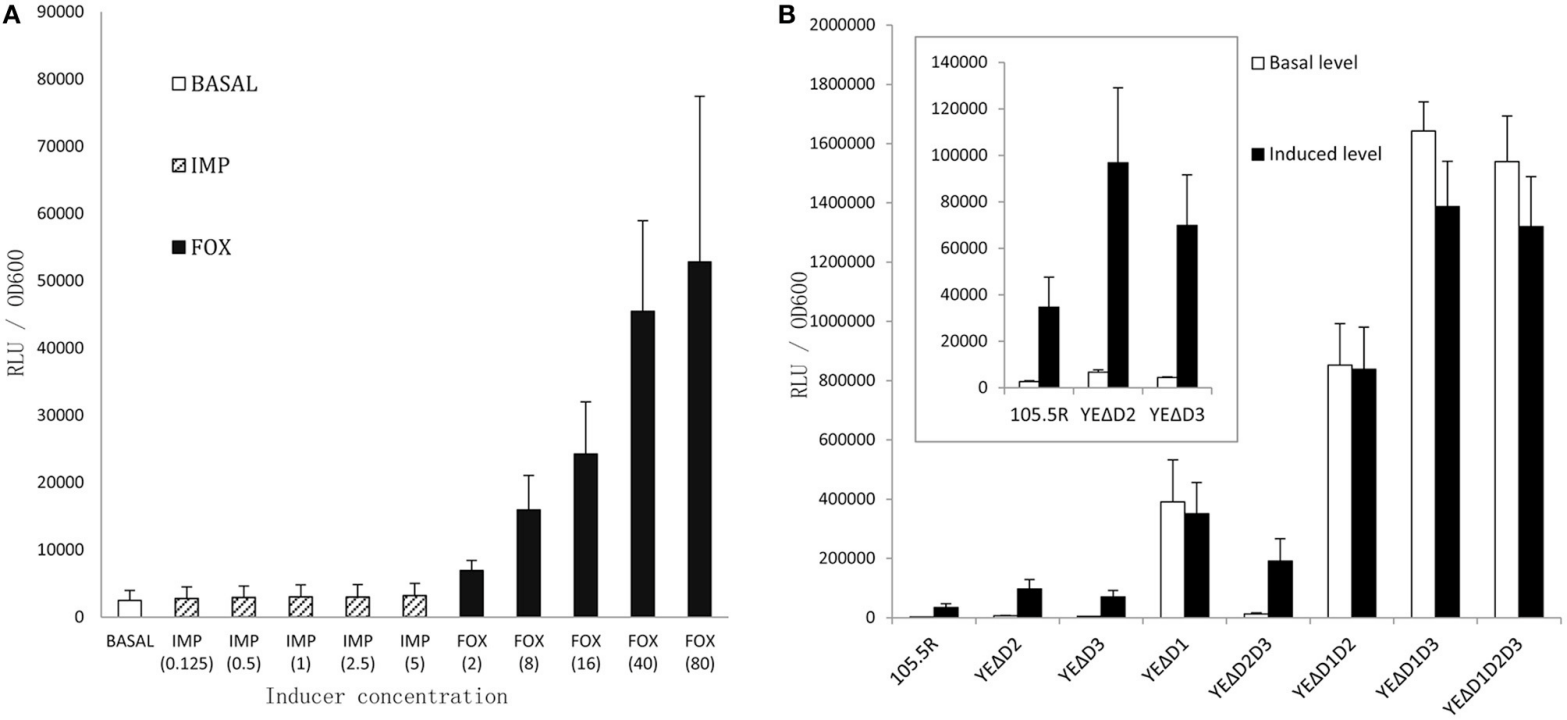
**Induction assay by monitor the *ampC* promoter activity of *Y. enterocolitica* 105.5R(r) to choose an appropriate inducer (A), each group was added in IMP or FOX by 0.125-, 1-, 2-, 5-, 10-fold MIC-value, respectively**. Then the activity of *ampC* promoter of 105.5R(r) and its seven derived mutants were tested **(B)**, the strains were grown in the 40 mg/L Cefoxitin for 1 h. Error bars indicate standard deviations for triplicate experiments.

As shown in Figure 3A, we confirmed the best inducer of the induction assay was cefoxitin (40 mg/L). Figure 3B showed inactivation of the *ampD* homologs caused three different levels of enhancements in *ampC* expression. At level one, mutant strains YEΔD2 (*ampD2* inactivation), YEΔD3 (*ampD3* inactivation), and the double mutant strain YEΔD2D3 (*ampD2*-*ampD3* double inactivation) only slightly increased *ampC* promoter activity. At level two, luminescence values showed an obvious elevation when the *ampD1* inactivated strain, YEΔD1, was examined; and hence, the *ampC* promoter activity of double mutant strain YEΔD1D2 was further enhanced. Finally, at level three, in *ampD1*–*ampD3* relative double/triple mutant strains, YEΔD1D3 and YEΔD1D2D3, *ampC* promoter activity was dramatically increased in both basal and induced conditions. But the activity was elevated only at modest level under the inducible conditions. At the same time, not surprisingly, neither any mutation nor inducer change the BlaA expression (data not shown).

### Role of AmpD1, AmpD2, and AmpD3 in β-lactamase activity and β-lactam resistance in *Y. enterocolitica*

**Figure 4:**
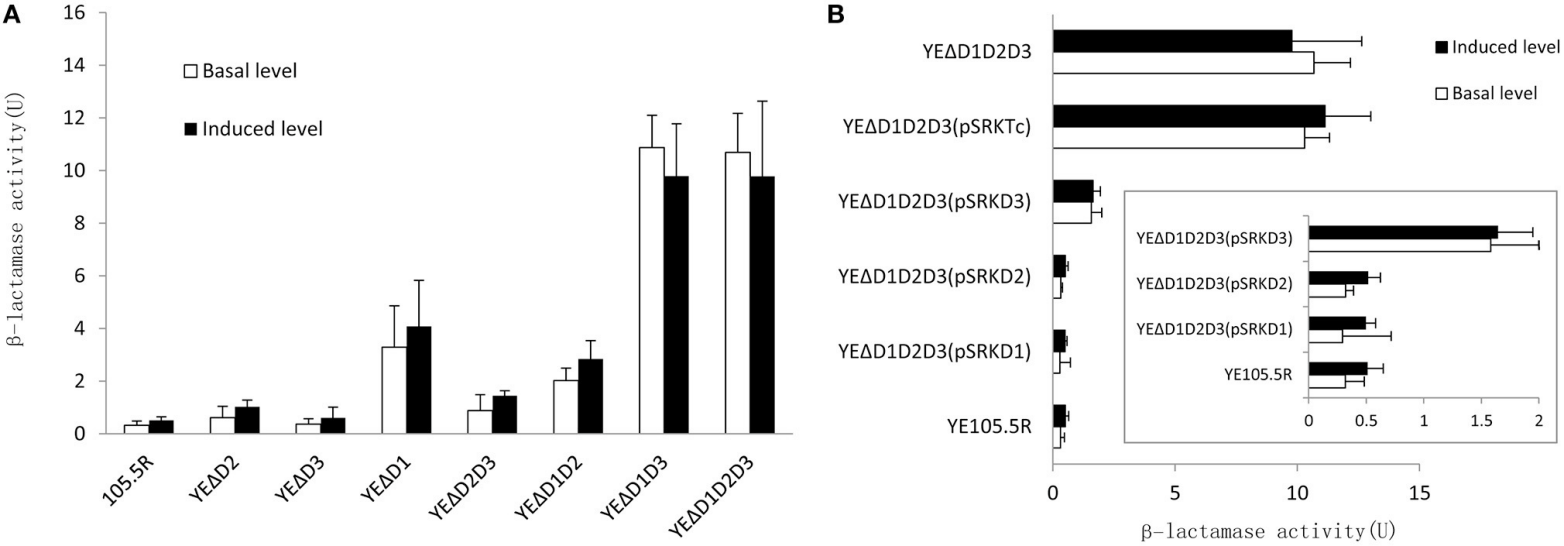
**Three AmpD homologs cooperate to regulate the *ampC* expression in *Y. enterocolitica* in eight test strains**. **(A)** Inactivation of AmpD homologs causes the elevation on β-lactamase activity in variant degree. **(B)** In complementation assay, hyperproduction of single AmpD1 (pSRKTcD1) and AmpD2 (pSRKTcD2) in YEΔD1D2D3 will reduce the β-lactamase activity into wild-type strain levels, however, single AmpD3 (pSRKTcD3) do not possess full capacity as AmpD1 and AmpD3. This data are the average of the measurements made in triplicate, the error bars indicate standard deviations.

To further understand the role of AmpD homologs in *Y. enterocolitica*, we tested the β-lactamase activity and the minimal inhibitory concentrations (MICs) of 15 antibiotics in the eight test strains mentioned above. As shown in Figure 4A, the value of β-lactamase activity in wild-type strain with or wothout inducer was consist with the biotype 3 strain reported by Stock et al. (2000). On the *ampD* mutant aspect, the levels of β-lactamase activity was consistent with luminescence values, three β-lactamase activity levels occur. At level one, β-lactamase activity of mutant strains YEΔD2 (*p* < 0.05), YEΔD2D3 (*p* < 0.05), and YEΔD3 (no statistically significant) was only slightly increased compared with the wild-type strain 105.5R(r) (~1.2- to 3-fold), whereas a modest level increase appeared in strain YEΔD1 (*p* < 0.05) and YEΔD1D2 (*p* < 0.05; ~10- to 20-fold) compared to at level two. Finally, at level three, complete derepression was reached in YEΔD1D3 (*p* < 0.05) and YEΔD1D2D3 (*p* < 0.05) where the β-lactamase activity of these two strains were significantly increased (~38-fold) compared to YE105.5R(r) and could not be further induced by cefoxitin (no statistically significant).

For β-lactam resistance, as shown in Figure 4A, three levels of AmpC hyper-production elevate the resistance capacity to most penicillins, first-generation cephalosporins, third-generation cephalosporins, monobactam, and cephamycins at the three different levels. At the same time, MICs of imipenem, meropenem, and ciprofloxacin were not changed. It should be noted that no CLSI breakpoint was surpassed in any of the antibiotics tested except for Ampicillin, Ticarcillin, and Cephazolin which were native for resistance in *Y. enterocolitica*.

The complementation assays were performed introducing pSRKTcD1, pSRKTcD2, and pSRKTcD3 into the triple-mutant strain YEΔD1D2D3. After quantifying the β-lactamase activity of each complementary strain, as shown in Figure 4B, YEΔD1D2D3 (pSRKTcD1) and YEΔD1D2D3 (pSRKTcD2) completely restored the β-lactamase activity to the level of the wild-type strain YE105.5R(r); while in YEΔD1D2D3 (pSRKTcD3), the β-lactamase activity significantly decreased, but not reaching the level of wild-type strain (*p* < 0.05).

## Disscussion

The biological function of AmpD was first characterized in *E. cloacae* and *C. freundii* (Lindberg et al., 1987; Peter et al., 1988) where it played an essential role in suppressing the *ampC* expression through the *ampR-ampC* system indirectly and later studies on AmpD in other Gram-negative organisms taken *Enterbacteraceae* as the paradigm (Juan et al., 2006; Yang et al., 2009). However, each *Enterobacteriaceae* members usually have peculiar features in AmpC regulation (Jacoby, 2009). In *E. coli*, AmpC production was not inducible because having no AmpR, and it was regulated by a promoter and attenuator mechanism (Jaurin et al., 1981). In addition, expression of *ampC* in *Acinetobacter baumannii* was also not inducible lacking AmpR (Bou and Martinez-Beltran, 2000). As a member of *Enterobacteriaceae, Y. enterocolitica* is not well-understood in the features of *ampC* regulation. To date, a three-step *ampC* regulation mechanism driven by three *ampD* homologs was only observed in *P. aeruginosa* (Juan et al., 2006), while two AmpD homologs were also identified in *Stenotrophomonas maltophilia* but only AmpDI was effective in β-lactamase regulation (Yang et al., 2009; Talfan et al., 2013).

In this study, we first reported the role of AmpD in *Y. enterocolitica* and there has three AmpD homologs with different features termed in AmpD1, AmpD2, and AmpD3 that coordinate to repress the expression of *ampC* β-lactamase. As shown in Figure 3, inactivation of *ampD1* (YEΔD1) results in an obvious increased in basal and inducible levels compared to the wild type (WT) strain, and a higher basal and inducible level was achieved in *ampD1–ampD2* double mutant strain (YEΔD1D2). However, although individual AmpD3 was ineffective (elevate the value but no statistical significance), the *ampD1*–*ampD3* double deletion strain YEΔD1D3 caused a significant increase from the basal expression level of *ampC* and cannot be further induced by imipemem. The full derepression phenotype was achieved in YEΔD1D3, suggesting a synergetic effect between AmpD1 and AmpD3. Finally, at the last level, triple mutants strain YEΔD1D2D3 was as same as YEΔD1D3, achieving the full derepression phenotype.

This three-step regulation mechanism has similarity with the stepwise regulation mechanism in *P. aeruginosa* with a few differences (Juan et al., 2006). In *P. aeruginosa*, over-expression of each of the three AmpD homologs can return the high-level expression back to wild-type strain levels, suggesting the three *ampD* homologs involved are repressing the *ampC* expression quantitatively rather than qualitatively. However, in *Y. enterocolitica*, after introducing recombinant expression vector pSRKTcD1 and pSRKTcD2 (high expression AmpD1 and AmpD2, respectively) to YEΔD1D2D3, the high β-lactamase activity was reduced to the low wild-type strain level. Compared with AmpD1 and AmpD2, solely AmpD3 did not possess the full capacity to repress the *ampC* expression. After introducing pSRKTcD3 (high expression AmpD3) into YEΔD1D2D3, the β-lactamase activity is a limited reduction, not returning to the level of the wild-type strain. Further, in *P. aeruginosa*, PAΔDDh3 shows a high-level hyper-inducible expression state not observed in *Y. enterocolitica*.

As shown in Table 3, the *ampD* mutant strains was maximal quadrupling the MIC-values on TIC, PIP, TZP, CFZ, CAZ, ATM but there is no CLSI breakpoint was surpassed in any of the antibiotics tested in this experiment. This consequence might seem like lack of clinical significance on the surface, but actually, *ampC*-*ampR* system may participate in many aspect of bacterial metabolization, as AmpR is a global transcriptional factor in β-lactamases, proteases, quorum sensing, virulence factors, and even iron acquisition (Balasubramanian et al., 2014; Caille et al., 2014). AmpD was also at the crossroads in cell-wall recycling and AmpC regulation simultaneously (Lee et al., 2009). Three AmpD homologs cooperated with each other in cell-wall recycling system results in a stabilized and efficient process to recycle the useless peptidoglycan degradation products into cell wall synthesis, so less energy will be needed in cell growth and division. In survival in eukaryotes, extracellular muropeptides can be recognized by the immune system, a potent cell-wall recycling system ensures less muropeptides release results in higher survival rates (Johnson et al., 2013). Moya et al. (2008) found in competition experiments in the mouse model *in vivo*, the *ampD ampDh2 ampDh3* and *ampC* quadruple mutant *P. aeruginosa* strain completely lost its biological competitiveness.

**Table 3:** **MICs of antibiotics for strain 105.5R(r) and *ampD* mutants**.

**Antibiotic**	**MIC (mg/ml) of antibiotic of strain^a^^,^^b^**							
	**YE105.5R(r)**	**YEΔD1**	**YEΔD2**	**YEΔD3**	**YEΔD1D2**	**YEΔD2D3**	**YEΔD1D2D3**	**YEΔD1D2D3**
**PENICILLINS**								
AMP	32	32	32	32	32	64	32	64
TIC	1	4	2	1	4	4	2	4
PIP	2	16	4	2	16	16	4	16
SAM	16	16	16	16	16	16	16	16
TZP	1	4	2	1	4	4	2	4
**CEPHALOSPORINS**								
CFZ	128	512	128	256	512	512	256	512
CAZ	0.25	1	0.5	0.25	1	2	0.25	2
FEP	0.25	0.25	0.25	0.25	0.25	0.25	0.25	0.25
CRO	≤ 0.125	0.25	0.25	0.25	0.5	0.5	0.25	0.5
**MONOBACTAM**								
ATM	≤ 0.125	0.5	≤ 0.125	≤ 0.125	0.5	1	0.25	1
**CEPHAMYCINS**								
CTT	2	4	2	2	4	4	4	4
FOX	8	8	8	8	8	8	8	8
**CARBAPENEMS**								
IPM	0.5	0.5	0.5	0.5	0.5	0.5	0.5	0.5
MEM	0.25	0.25	0.25	0.25	0.25	0.25	0.25	0.25
**QUINOLONES**								
CIP	≤ 0.03	≤ 0.03	≤ 0.03	≤ 0.03	≤ 0.03	≤ 0.03	≤ 0.03	≤ 0.03

a *AMP, Ampicillin; TIC, Ticarcillin; PIP, Piperacillin; SAM, Ampicillin-sulbactam; TZP, Piperacillin-tazobactam; CFZ, Cefazolin; CAZ, Ceftazidime; FEP, Cefepime; CRO, Ceftriaxone; ATM, Aztreonam; CTT, Cefotetan; FOX, Cefoxitin; IPM, Imipenem; MEM, Meropenem; CIP, Ciprofloxacin*.

b *MIC was determined in triplicate by standard two-fold serial broth microdilution method, all measurements were performed in triplicate*.

Recent studies demonstrate *Y. enterocolitica* strains of specific biovars tend to display similar features in expression and the activities of two different β-lactamases (Pham et al., 2000; Stock et al., 2000; Sharma et al., 2006). Further studies are necessary to understand the distribution and function of AmpD homologs in each biovar of the *Y. enterocolitica* strains. Especially the AmpD3, less effective alone, but has a potent synergetic effect together with AmpD1. Furthermore, recent studies showed that penicillin-binding protein 4 (DacB) affected the AmpC expression in some bacteria (Zamorano et al., 2010), it will be attractive to understand its function in *Y. enterocolitica*.

In summary, in the present study, we first illustrate the role of AmpD in *Y. enterocolitica* and a three-step regulation mechanism of *ampC* expression was found. Furthermore, this was also the first report such complicated *ampC* regulation mechanism appears in *Enterobacteriaceae*. It will be very interesting to examine if this minority mechanism appears in other *Enterobacteriaceae* family strains.

## Author contributions

HJ, SS, and ChaL design the experiment together. ChaL, YC, XL, ChuL, and HH finish the work. JL, RD, and JZ participate in the manuscript translation.

### Conflict of interest statement

The authors declare that the research was conducted in the absence of any commercial or financial relationships that could be construed as a potential conflict of interest.

## References

[B1] Balasubramanian D. Kumari H. Jaric M. Fernandez M. Turner K. H. Dove S. L.. (2014). Deep sequencing analyses expands the *Pseudomonas aeruginosa* AmpR regulon to include small RNA-mediated regulation of iron acquisition, heat shock and oxidative stress response. Nucleic Acids Res. 42, 979–998. 10.1093/nar/gkt942 24157832 PMC3902932

[B2] Bent Z. W. Young G. M. (2010). Contribution of BlaA and BlaB beta-lactamases to antibiotic susceptibility of *Yersinia enterocolitica* biovar 1B. Antimicrob. Agents Chemother. 54, 4000–4002. 10.1128/AAC.01754-09 20547799 PMC2935026

[B3] Bou G. Martínez-Beltrán J. (2000). Cloning, nucleotide sequencing, and analysis of the gene encoding an AmpC beta-lactamase in *Acinetobacter baumannii*. Antimicrob. Agents Chemother. 44, 428–432. 10.1128/AAC.44.2.428-432.2000 10639377 PMC89698

[B4] Caille O. Zincke D. Merighi M. Balasubramanian D. Kumari H. Kong K. F.. (2014). Structural and functional characterization of *Pseudomonas aeruginosa* global regulator AmpR. J. Bacteriol. 196, 3890–3902. 10.1128/JB.01997-14 25182487 PMC4248820

[B5] Cheng Q. Park J. T. (2002). Substrate specificity of the AmpG permease required for recycling of cell wall anhydro-muropeptides. J. Bacteriol. 184, 6434–6436. 10.1128/JB.184.23.6434-6436.2002 12426329 PMC135433

[B6] Cornelis G. Abraham E. P. (1975). Beta-lactamases from *Yersinia enterocolitica*. J. Gen. Microbiol. 87, 273–284. 10.1099/00221287-87-2-273 237976

[B7] De La Prieta M. C. Francia M. V. Seoane A. Lobo J. M. (2006). Characterization of defective beta-lactamase genes in *Yersinia enterocolitica*. J. Antimicrob. Chemother. 58, 661–664. 10.1093/jac/dkl267 16807253

[B8] De La Prieta M. C. Seoame A. Díaz J. Navas J. Garciá-Lobo J. M. (1995). Beta-lactamase genes and beta-lactamic susceptibility in *Yersinia enterocolitica*. Contrib. Microbiol. Immunol. 13, 184–187. 8833829

[B9] Généreux C. Dehareng D. Devreese B. Van Beeumen J. Frère J. M. Joris B. (2004). Mutational analysis of the catalytic centre of the *Citrobacter freundii* AmpD N-acetylmuramyl-L-alanine amidase. Biochem. J. 377, 111–120. 10.1042/bj20030862 14507260 PMC1223845

[B10] Hammer B. K. Bassler B. L. (2007). Regulatory small RNAs circumvent the conventional quorum sensing pathway in pandemic *Vibrio cholerae*. Proc. Natl. Acad. Sci. U.S.A. 104, 11145–11149. 10.1073/pnas.0703860104 17556542 PMC1888797

[B11] Hanson N. D. Sanders C. C. (1999). Regulation of inducible AmpC beta-lactamase expression among Enterobacteriaceae. Curr. Pharm. Des. 5, 881–894. 10539994

[B12] Höltje J. V. Kopp U. Ursinus A. Wiedemann B. (1994). The negative regulator of beta-lactamase induction AmpD is a N-acetyl-anhydromuramyl-L-alanine amidase. FEMS Microbiol. Lett. 122, 159–164. 10.1111/j.1574-6968.1994.tb07159.x 7958768

[B13] CLSI (2015). Performance Standards for Antimicrobial Susceptibility Testing. Twenty-fourth Informational Supplement. CLSI Document M100-S25. Wayne, PA.

[B14] Jacobs C. Frère J. M. Normark S. (1997). Cytosolic intermediates for cell wall biosynthesis and degradation control inducible beta-lactam resistance in gram-negative bacteria. Cell 88, 823–832. 10.1016/S0092-8674(00)81928-5 9118225

[B15] Jacobs C. Huang L. J. Bartowsky E. Normark S. Park J. T. (1994). Bacterial cell wall recycling provides cytosolic muropeptides as effectors for beta-lactamase induction. EMBO J. 13, 4684–4694. 7925310 10.1002/j.1460-2075.1994.tb06792.x PMC395403

[B16] Jacobs C. Joris B. Jamin M. Klarsov K. Van Beeumen J. Mengin-Lecreulx D.. (1995). AmpD, essential for both beta-lactamase regulation and cell wall recycling, is a novel cytosolic N-acetylmuramyl-L-alanine amidase. Mol. Microbiol. 15, 553–559. 10.1111/j.1365-2958.1995.tb02268.x 7783625

[B17] Jacoby G. A. (2009). AmpC beta-lactamases. Clin. Microbiol. Rev. 22, 161–182, Table of Contents. 10.1128/CMR.00036-08 19136439 PMC2620637

[B18] Jaurin B. Grundstrom T. Edlund T. Normark S. (1981). The *E. coli* beta-lactamase attenuator mediates growth rate-dependent regulation. Nature 290, 221–225. 10.1038/290221a0 7010184

[B19] Johnson J. W. Fisher J. F. Mobashery S. (2013). Bacterial cell-wall recycling. Ann. N. Y. Acad. Sci. 1277, 54–75. 10.1111/j.1749-6632.2012.06813.x 23163477 PMC3556187

[B20] Juan C. Moyá B. Pérez J. L. Oliver A. (2006). Stepwise upregulation of the *Pseudomonas aeruginosa* chromosomal cephalosporinase conferring high-level beta-lactam resistance involves three AmpD homologues. Antimicrob. Agents Chemother. 50, 1780–1787. 10.1128/AAC.50.5.1780-1787.2006 16641450 PMC1472203

[B21] Kerff F. Petrella S. Mercier F. Sauvage E. Herman R. Pennartz A.. (2010). Specific structural features of the N-acetylmuramoyl-L-alanine amidase AmiD from *Escherichia coli* and mechanistic implications for enzymes of this family. J. Mol. Biol. 397, 249–259. 10.1016/j.jmb.2009.12.038 20036252

[B22] Kong K. F. Jayawardena S. R. Indulkar S. D. Del Puerto A. Koh C. L. Høiby N. (2005). *Pseudomonas aeruginosa* AmpR is a global transcriptional factor that regulates expression of AmpC and PoxB beta-lactamases, proteases, quorum sensing, and other virulence factors. Antimicrob. Agents Chemother. 49, 4567–4575. 10.1128/AAC.49.11.4567-4575.2005 16251297 PMC1280116

[B23] Lee M. Zhang W. Hesek D. Noll B. C. Boggess B. Mobashery S. (2009). Bacterial AmpD at the crossroads of peptidoglycan recycling and manifestation of antibiotic resistance. J. Am. Chem. Soc. 131, 8742–8743. 10.1021/ja9025566 19496566 PMC2845155

[B24] Liang J. Wang X. Xiao Y. Cui Z. Xia S. Hao Q.. (2012). Prevalence of *Yersinia enterocolitica* in pigs slaughtered in Chinese abattoirs. Appl. Environ. Microbiol. 78, 2949–2956. 10.1128/AEM.07893-11 22327599 PMC3318836

[B25] Lindberg F. Lindquist S. Normark S. (1987). Inactivation of the ampD gene causes semiconstitutive overproduction of the inducible *Citrobacter freundii* beta-lactamase. J. Bacteriol. 169, 1923–1928. 3032901 10.1128/jb.169.5.1923-1928.1987 PMC212046

[B26] Moya B. Juan C. Albertí S. Pérez J. L. Oliver A. (2008). Benefit of having multiple ampD genes for acquiring beta-lactam resistance without losing fitness and virulence in *Pseudomonas aeruginosa*. Antimicrob. Agents Chemother. 52, 3694–3700. 10.1128/AAC.00172-08 18644952 PMC2565882

[B27] Park J. T. Uehara T. (2008). How bacteria consume their own exoskeletons (turnover and recycling of cell wall peptidoglycan). Microbiol. Mol. Biol. Rev. 72, 211–227, Table of Contents. 10.1128/MMBR.00027-07 18535144 PMC2415748

[B28] Peter K. Korfmann G. Wiedemann B. (1988). Impact of the ampD gene and its product on beta-lactamase production in *Enterobacter cloacae*. Rev. Infect. Dis. 10, 800–805. 10.1093/clinids/10.4.800 3263688

[B29] Petersen T. N. Brunak S. Von Heijne G. Nielsen H. (2011). SignalP 4.0: discriminating signal peptides from transmembrane regions. Nat. Methods 8, 785–786. 10.1038/nmeth.1701 21959131

[B30] Pham J. N. Bell S. M. Martin L. Carniel E. (2000). The beta-lactamases and beta-lactam antibiotic susceptibility of *Yersinia enterocolitica*. J. Antimicrob. Chemother. 46, 951–957. 10.1093/jac/46.6.951 11102414

[B31] Philippe N. Alcaraz J. P. Coursange E. Geiselmann J. Schneider D. (2004). Improvement of pCVD442, a suicide plasmid for gene allele exchange in bacteria. Plasmid 51, 246–255. 10.1016/j.plasmid.2004.02.003 15109831

[B32] Seoane A. Francia M. V. García Lobo J. M. (1992). Nucleotide sequence of the ampC-ampR region from the chromosome of *Yersinia enterocolitica*. Antimicrob. Agents Chemother. 36, 1049–1052. 10.1128/AAC.36.5.1049 1510392 PMC188833

[B33] Sharma S. Mittal S. Mallik S. Virdi J. S. (2006). Molecular characterization of beta-lactamase genes blaA and blaB of *Yersinia enterocolitica* biovar 1A. FEMS Microbiol. Lett. 257, 319–327. 10.1111/j.1574-6968.2006.00191.x 16553870

[B34] Simon R. Priefer U. Puhler A. (1983). A broad range mobilization system for *in vivo* genetic engineering: transposon mutagenesis in gramnegative bacteria. Nat. BioTechnol. 1, 784–791. 10.1038/nbt1183-784

[B35] Stock I. Heisig P. Wiedemann B. (1999). Expression of beta-lactamases in *Yersinia enterocolitica* strains of biovars 2, 4 and 5. J. Med. Microbiol. 48, 1023–1027. 10.1099/00222615-48-11-1023 10535647

[B36] Stock I. Heisig P. Wiedemann B. (2000). Beta-lactamase expression in *Yersinia enterocolitica* biovars 1A, 1B, and 3. J. Med. Microbiol. 49, 403–408. 10.1099/0022-1317-49-5-403 10798551

[B37] Talfan A. Mounsey O. Charman M. Townsend E. Avison M. B. (2013). Involvement of mutation in ampD I, mrcA, and at least one additional gene in beta-lactamase hyperproduction in *Stenotrophomonas maltophilia*. Antimicrob. Agents Chemother. 57, 5486–5491. 10.1128/AAC.01446-13 23979761 PMC3811264

[B38] Wang X. Cui Z. Jin D. Tang L. Xia S. Wang H.. (2009). Distribution of pathogenic *Yersinia enterocolitica* in China. Eur. J. Clin. Microbiol. Infect. Dis. 28, 1237–1244. 10.1007/s10096-009-0773-x 19575249

[B39] Wang X. Li Y. Jing H. Ren Y. Zhou Z. Wang S. (2011). Complete genome sequence of a *Yersinia enterocolitica* “Old World” (3/O:9) strain and comparison with the “New World” (1B/O:8) strain. J. Clin. Microbiol. 49, 1251–1259. 10.1128/JCM.01921-10 21325549 PMC3122819

[B40] Yang T. C. Huang Y. W. Hu R. M. Huang S. C. Lin Y. T. (2009). AmpDI is involved in expression of the chromosomal L1 and L2 beta-lactamases of *Stenotrophomonas maltophilia*. Antimicrob. Agents Chemother. 53, 2902–2907. 10.1128/AAC.01513-08 19414581 PMC2704650

[B41] Zamorano L. Reeve T. M. Deng L. Juan C. Moyá B. Cabot G.. (2010). NagZ inactivation prevents and reverts beta-lactam resistance, driven by AmpD and PBP 4 mutations, in *Pseudomonas aeruginosa*. Antimicrob. Agents Chemother. 54, 3557–3563. 10.1128/AAC.00385-10 20566764 PMC2934985

[B42] Zhou Y. Y. Zhang H. Z. Liang W. L. Zhang L. J. Zhu J. Kan B. (2013). Plasticity of regulation of mannitol phosphotransferase system operon by CRP-cAMP complex in *Vibrio cholerae*. Biomed. Environ. Sci. 26, 831–840. 10.3967/bes2013.006 24215877

